# Quantifying the efficiency of CO_2_ capture by Lewis pairs†
†Electronic supplementary information (ESI) available. See DOI: 10.1039/c6sc05607e


**DOI:** 10.1039/c6sc05607e

**Published:** 2017-02-20

**Authors:** Jay J. Chi, Timothy C. Johnstone, Dan Voicu, Paul Mehlmann, Fabian Dielmann, Eugenia Kumacheva, Douglas W. Stephan

**Affiliations:** a Department of Chemistry , University of Toronto , 80 St. George St. , Toronto , Ontario M5S 3H6 , Canada . Email: dstephan@chem.utoronto.ca; b Institut für Anorganische und Analytische Chemie , Westfälische Wilhelms-Universität Münster , Corrensstrasse 30 , 48149 Münster , Germany

## Abstract

A microfluidic strategy has been used for the time- and labour-efficient evaluation of the relative efficiency and thermodynamic parameters of CO_2_ binding by three Lewis acid/base combinations, where efficiency is based on the amount of CO_2_ taken up per binding unit in solution. Neither *t*Bu_3_P nor B(C_6_F_5_)_3_ were independently effective at CO_2_ capture, and the combination of the imidazolin-2-ylidenamino-substituted phosphine (NI*i*Pr)_3_P and B(C_6_F_5_)_3_ was equally ineffective. Nonetheless, an archetypal frustrated Lewis pair (FLP) comprised of *t*Bu_3_P and B(C_6_F_5_)_3_ was shown to bind CO_2_ more efficiently than either the FLP derived from tetramethylpiperidine (TMP) and B(C_6_F_5_)_3_ or the highly basic phosphine (NI*i*Pr)_3_P. Moreover, the proposed microfluidic platform was used to elucidate the thermodynamic parameters for these reactions.

## Abstract 2

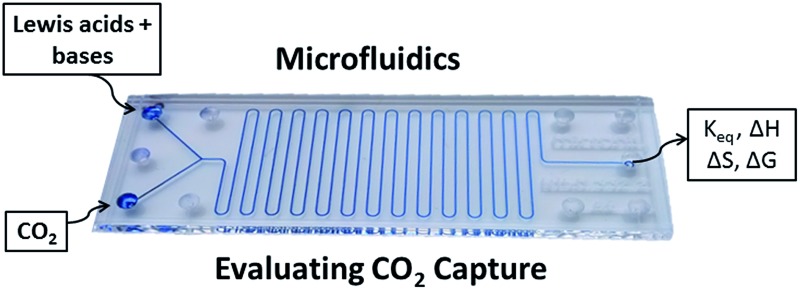
A microfluidic strategy is used to assess the relative efficiency and thermodynamic parameters of CO_2_ binding by three Lewis acid/base combinations.

## Introduction

Anthropogenic carbon dioxide (CO_2_) emissions continue to climb to unprecedented levels and have played a key role in global climate change.^1^ This worldwide issue has prompted many researchers to explore a wide variety of approaches to both reduce CO_2_ emissions and lower CO_2_ concentrations in the atmosphere. Efforts targeting the use of CO_2_ as a C_1_ chemical feedstock for conversion to formic acid, carbon monoxide,^2^ or reusable fuels such as methane or methanol,^3^ have prompted many studies targeting new catalyst development.^4^ Although these developments offer the potential for disruptive technologies, it is important to note that the capture of CO_2_ will be an integral component of any such advancement. A variety of approaches have been explored to capture CO_2_ including the use of zeolites, silica gels, aluminas, and activated carbons,^5^ as well as sophisticated metal–organic frameworks (MOFs).^6^

Investigations of the reactions of CO_2_ with main group reagents have included a variety of amines,^7^ alkanolamines^8^ amidines, guanidines,^9^ and *N*-heterocyclic carbenes (NHCs).^10^ A decade ago, the use of frustrated Lewis pairs (FLPs) to capture CO_2_ emerged with the report of Stephan, Erker, and coworkers who described intramolecular and intermolecular B/P-based FLPs for the capture of CO_2_.^11^ Since then, a wide variety of B/N,^12^ B/P,^13^ Al/P,^14^ and Si/P^12a,15^ systems have been shown to capture or effect stoichiometric or catalytic reduction of CO_2_. In a very recent development, Dielmann and coworkers described the synthesis of highly basic phosphines, generated by the inclusion of imidazolin-2-ylidenamino substituents.^16^ These are the first phosphines to be shown to sequester CO_2_ in the absence of the Lewis acid necessary to form an FLP.^17^

Although a number of FLP and main group systems have been shown to capture CO_2_,^13*c*^ the ability to quantitatively compare the efficiency of such systems remains experimentally challenging. Standard batch-scale characterization methods for reactions at the CO_2_ gas–liquid interface suffer from long reaction times and are often diffusion controlled.^18^ Recently, Kumacheva and coworkers developed a microfluidic (MF) platform for the study of gas/liquid reactions.^19^ The MF methodology was validated for the well-studied CO_2_ reaction with amine^19*b*^ and used small amounts of reagents thus providing fast and cost-efficient access to thermodynamic data for gas/liquid reactions (10–15 min per experiment).

**Fig. 1:**
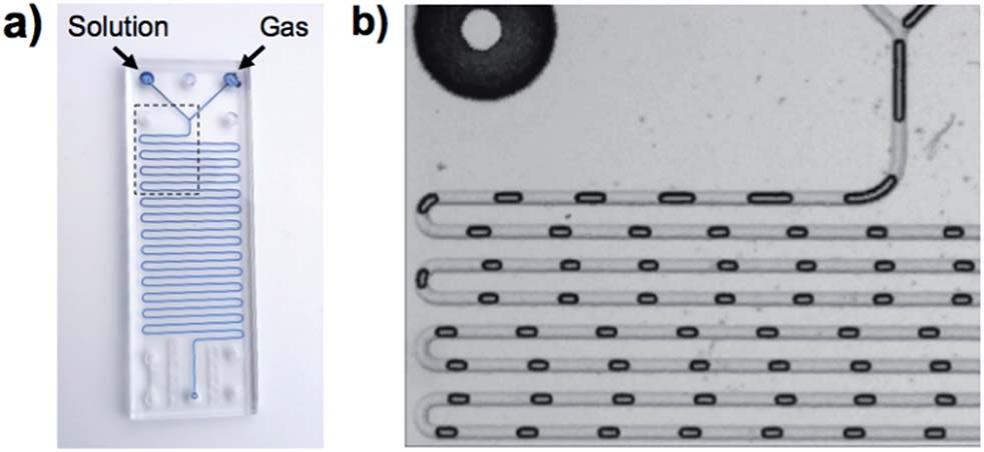
(a) Schematic depiction of the MF gas/liquid device. (b) Magnified view of the outlined region shown in (a), which shows the shrinkage of the gas plugs as they flow through the channel.

In Fig. 1, a gas and a reagent solution are supplied to two inlets of a MF reactor. At a Y-junction, the gaseous stream breaks up in a periodic manner to generate uniformly sized gas plugs that are separated by liquid segments (slugs). As alternating gas plugs and solution slugs flow through the MF channel, the dissolution of the gas and its reaction with reagents in the solution results in a decrease in the volume of gas plugs with time (or the distance from the Y-junction). Analysis of digitized images of the gas plugs allows for the quantification of gas consumption using the ideal gas law.^19a,20^ After a particular time (directly related to distance in the MF reactor), the dissolved reagents and the gas reach equilibrium, and the gaseous plug volume remains constant. This enables the determination of the equilibrium constant of the reaction, and a study of the reaction at different temperatures enables assessment of the thermodynamic parameters, Δ*G*°, Δ*H*°, and Δ*S*°. The validity of this methodology was demonstrated with the study of the sequestration of CO_2_ by the FLP, ClB(C_6_F_5_)_2_/*t*Bu_3_P.^19a,20^

**Scheme 1:**
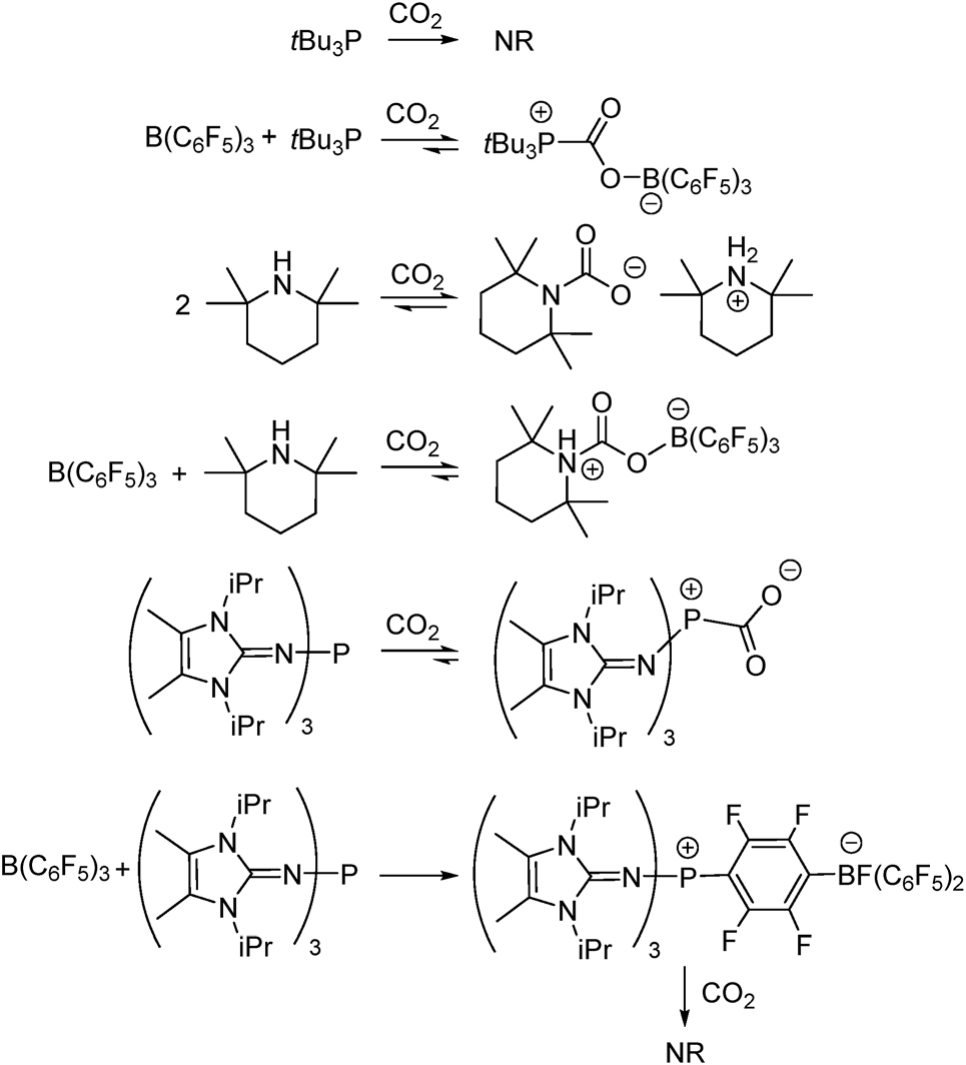
Reactions of Lewis acid–base combinations with CO_2_. NR = no reaction.

In the present work, this innovative MF approach has been applied to compare the efficiency of CO_2_ sequestration in reactions of three Lewis acid–base combinations with CO_2_. The prototypical FLP *t*Bu_3_P/B(C_6_F_5_)_3_, as well as the FLP derived from tetramethylpiperidine (TMP)/B(C_6_F_5_)_3_, were investigated. In addition, the extremely basic imidazolin-2-ylidenamino-substituted phosphine (NI*i*Pr)_3_P was investigated alone and in combination with B(C_6_F_5_)_3_. Although each of these systems is known to bind CO_2_ (Scheme 1),^11,12b,17b^ the present MF study provides qualitative and quantitative comparisons of this CO_2_ binding. Such data afford insights that are important for the design of main group systems for CO_2_ capture.

## Results and discussion

**Fig. 2:**
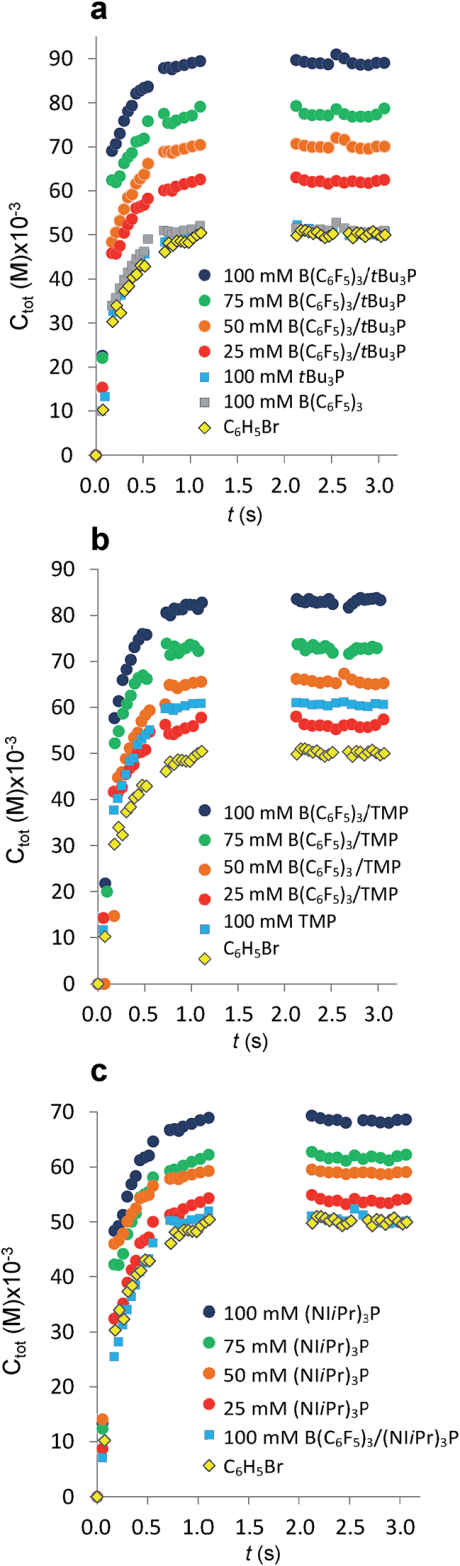
Variation in total concentration of CO_2_ transferred at 293 K from gas plugs to reagent solution slugs plotted as a function of time. The gaps in the data from 1.5 s to 2.0 s result from the exclusion of microchannel bends outside the region of interest (see ESI, Fig. S1†). (a) Plots for the FLP derived from *t*Bu_3_P and B(C_6_F_5_)_3_. (b) Plots for the FLP derived from TMP and B(C_6_F_5_)_3_. (c) Plots for the combination of (NI*i*Pr)_3_P and B(C_6_F_5_)_3_. For C_6_H_5_Br alone (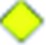), *C*_tot_ = [CO_2_]_dissolved_. Each experimental point represents the average of three experiments conducted under identical conditions, where 300 images were acquired for each experiments with a minimum of 4000 CO_2_ plugs.

The established MF protocol^19*a*^ was used to determine the thermodynamic parameters associated with the reaction of CO_2_ with combinations of the Lewis acid B(C_6_F_5_)_3_ and one of the Lewis bases, *t*Bu_3_P, TMP, or (NI*i*Pr)_3_P. Bromobenzene was selected as a suitable solvent due to its low volatility and the solubility of the reagents and corresponding CO_2_ adducts. In an initial reference experiment, physical dissolution of CO_2_ gas in bromobenzene was characterized by the temporal variation in the concentration of physically dissolved CO_2_ (*i.e.*, [CO_2_]_dissolved_) by analysing the digitized dimensions of alternating slugs of solvent and plugs of CO_2_ flowing through the MF reactor. By monitoring the decrease in CO_2_ plug volume and applying the ideal gas law^19*a*^ (eqn (1) and (2), see ESI, Fig. S1 and S2†), the number of moles of CO_2_ transferred from the gas plug to the adjacent liquid slug at time *t*, *n*_CO_2__(*t*), was determined. The equilibrium concentration of physically dissolved CO_2_ (*C*_tot_) was reached after approximately 2 s (Fig. 2).1
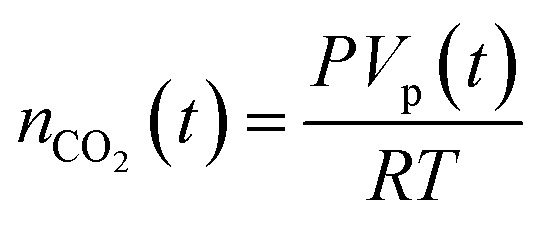

2
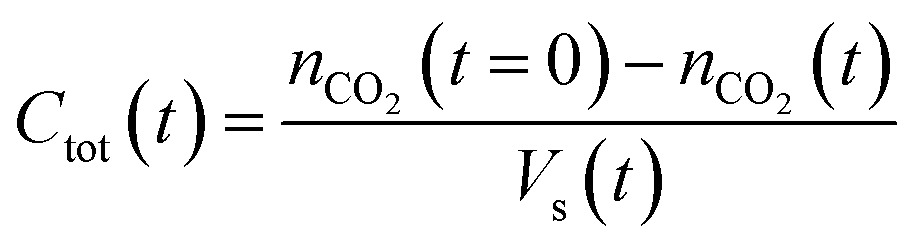

*n*_CO_2__(*t*): moles of CO_2_ in the plug at time *t*; *P*: pressure; *V*_p_(*t*): volume of CO_2_ at time *t*, *R*: gas constant (8.314 J mol^–1^ K^–1^); *T*: temperature; *V*_s_(*t*): volume of the liquid solution at time *t*.

The addition of either B(C_6_F_5_)_3_, or *t*Bu_3_P independently to the solvent had little effect on the equilibrium concentration of CO_2_ in the liquid slugs, beyond the dissolution of CO_2_ in bromobenzene (Fig. 2a). However, combining B(C_6_F_5_)_3_ and *t*Bu_3_P in solution resulted in increased CO_2_ uptake, which further increased with elevated FLP concentration. These observations are consistent with the known inability of the individual components to capture CO_2_, and the established efficacy with which CO_2_ is captured by this FLP. These observations are also consistent with our earlier MF study of CO_2_ capture by the related ClB(C_6_F_5_)_2_/*t*Bu_3_P FLP.^19*a*^

Investigation of the FLP derived from B(C_6_F_5_)_3_ and TMP revealed that TMP alone in solution is able to sequester CO_2_ (Fig. 2b, 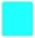), consistent with the known ability of secondary amines to reversibly bind CO_2_.^12*b*^ It is noteworthy, however, that the concurrent presence of B(C_6_F_5_)_3_ in solution results in a significantly enhanced CO_2_ uptake. Again, increasing concentration of the FLP results in increased CO_2_ sequestration.

**Fig. 3:**
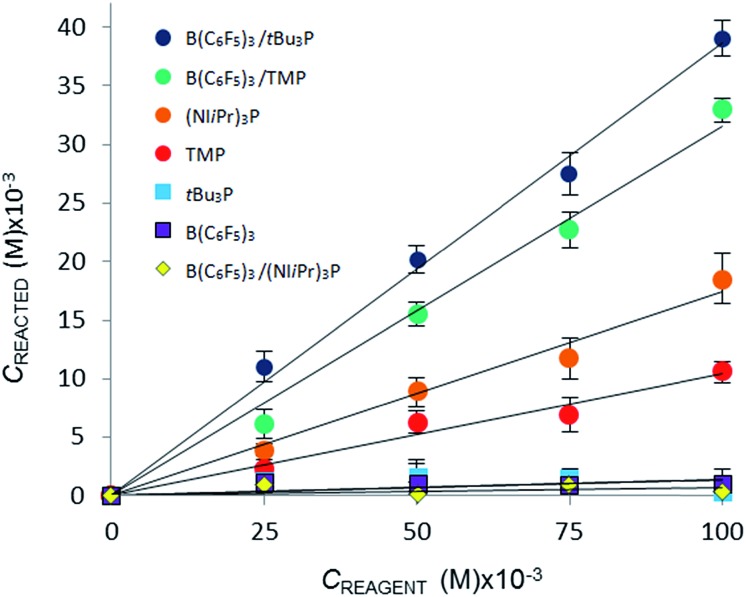
Variation in equilibrium concentration of CO_2_ (*C*_reacted_) plotted as a function of initial reagent concentration at *T* = 293 K (repeated in triplicate, analysing 300 images with a range of 4000–7000 plugs of CO_2_).

In sharp contrast to *t*Bu_3_P, increasing concentrations of (NI*i*Pr)_3_P led to increasing capture of CO_2_ (Fig. 3). These observations are consistent with the work of Dielmann and coworkers^17*b*^ who have demonstrated the ability of imidazolin-2-ylidenamino-substituted phosphines to bind CO_2_. In further contrast, addition of B(C_6_F_5_)_3_ to solutions of (NI*i*Pr)_3_P inhibited CO_2_ uptake beyond the physical dissolution of CO_2_ into the solvent (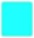 *vs.* 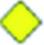, Fig. 2c). This result indicates an irreversible reaction of (NI*i*Pr)_3_P with B(C_6_F_5_)_3_. Monitoring of this reaction by NMR spectroscopy supports the formation of several products, including the zwitterionic product (NI*i*Pr)_3_PC_6_F_4_BF(C_6_F_5_)_2_ as the major species (Scheme 1, see ESI†). Analogous products have been previously reported for sterically encumbered, basic phosphines.^21^ Presumably, the highly basic nature of the phosphine (NI*i*Pr)_3_P prompts this reactivity with B(C_6_F_5_)_3_ and precludes capture of CO_2_.

The CO_2_ uptake caused directly by chemical reaction, *C*_reacted_, was determined by subtracting the [CO_2_]_dissolved_ (for the CO_2_–bromobenzene system) from the total equilibrium uptake of CO_2_, C_tot_, for each reagent. A plot of *C*_reacted_ against reagent concentration illustrates the relative efficacy of the reaction of CO_2_ with the Lewis acid, Lewis base, or Lewis acid–base combination (Fig. 3).

Using the concentration data allows determination of the equilibrium constants (*K*_eq_) for each system. In eqn (3), [CO_2_ adduct] is equal to *C*_reacted_ (determined from Fig. 3), the [Lewis acid] and [Lewis base] are calculated directly by subtracting *C*_reacted_ from the initial reagent concentrations, and [CO_2_]_dissolved_ is the equilibrium concentration of CO_2_ dissolved in bromobenzene (Fig. 2). In this fashion, the room temperature equilibrium constants for CO_2_ binding for *t*Bu_3_P/B(C_6_F_5_)_3_, TMP/B(C_6_F_5_)_3_, and (NI*i*Pr)_3_P were determined and are collected in Table 1. The data shown in Fig. 3 reveal that the FLP systems derived from *t*Bu_3_P or TMP with B(C_6_F_5_)_3_ are more efficient at CO_2_ capture by 29% and 16%, respectively, than the highly basic phosphine, (NI*i*Pr)_3_P. In this context, efficiency is taken to be the amount of CO_2_, per binding unit, sequestered by the binding reagents. These data also illustrate that the FLP *t*Bu_3_P/B(C_6_F_5_)_3_ is 11% more efficient at CO_2_ uptake than the FLP derived from TMP/B(C_6_F_5_)_3_. Thus, although previously reported NMR experiments demonstrated the ability of these systems to bind CO_2_, the present MF methodology provides a fast, efficient and high-throughput platform for quantitative ranking of the ability of these systems to bind CO_2_ at ambient temperature.

**Table 1:** Thermodynamic parameters for CO_2_ capture determined by the MF method^*a*^

Reagents	*K*_eq (293 K)_	Δ*H*_293_ ^*b*^ kJ mol^–1^	Δ*S*_293_ J mol^–1^ K^–1^	Δ*G*_293_ kJ mol^–1^
*t*Bu_3_P/B(C_6_F_5_)_2_Cl^19*a*^	223 M^–2^	–39.3	–89.3	–14.8
*t*Bu_3_P/B(C_6_F_5_)_3_	517 M^–2^	–100.0	–289.3	–15.2
TMP/B(C_6_F_5_)_3_	267 M^–2^	–73.8	–205.4	–13.6
(NI*i*Pr)_3_P	4158 M^–1^	–29.1	–30.8	–20.0

^*a*^Additional values for 273 K, 283 K, 303 K, and 313 K are deposited in the ESI (Table S2–S4).

^*b*^The value for Δ*H* is determined by the corresponding slope in Fig. 5.

**Fig. 4:**
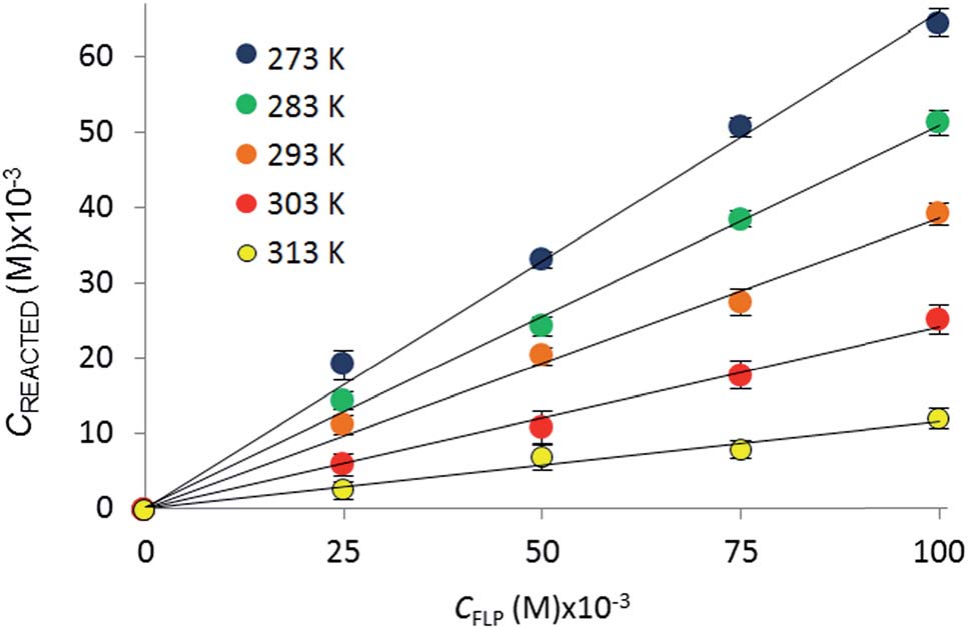
Variation in the equilibrium concentration of captured CO_2_, plotted as a function of initial FLP (B(C_6_F_5_)_3_ and *t*Bu_3_P) concentration at *T* = 273 K, 283 K, 293 K, 303 K, and 313 K (repeated in triplicate analysing 300 images with a range of 4000–7000 plugs of CO_2_) (see ESI† for plots for the other systems).

**Fig. 5:**
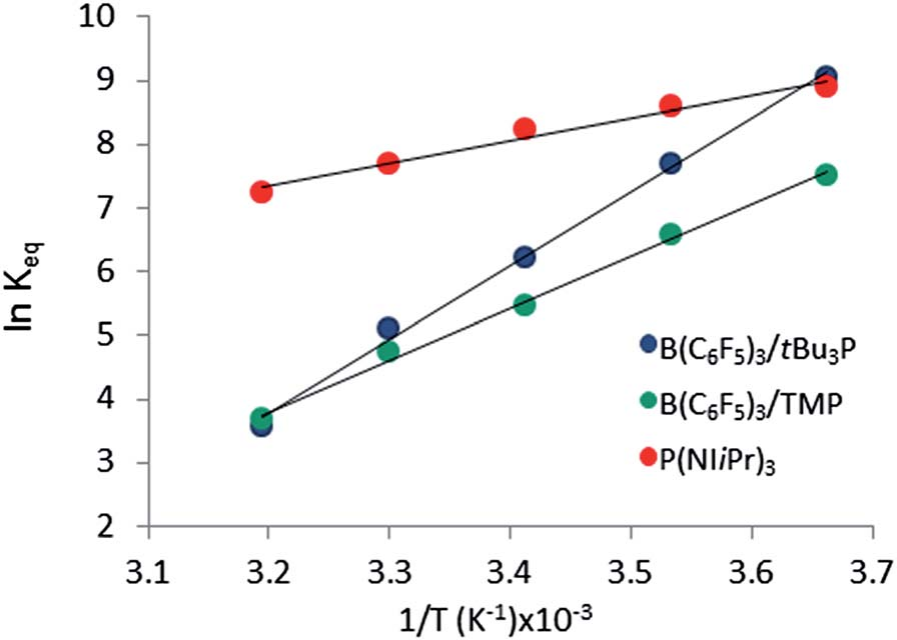
Plot of ln(*K*_eq_) *vs.* 1/*T* (*T* ranging from 273 K to 313 K).

The reactions of *t*Bu_3_P/B(C_6_F_5_)_3_, TMP/B(C_6_F_5_)_3_, and (NI*i*Pr)_3_P with CO_2_ were also studied in the temperature range from 273 to 313 K. A plot of the amount of CO_2_ captured (*C*_reacted_) *versus* the concentration of either the FLPs or (NI*i*Pr)_3_P was monotonic and linear. Due to the exothermic nature of the reactions,^19a,20^ as the reaction systems were cooled, the degree of CO_2_ capture was enhanced at each of the concentrations of reagents (Fig. 4). Using the values of *K*_eq_ at different temperatures, the corresponding Gibbs free energy for each reaction can be obtained (eqn (4)). The use of a van't Hoff plot (Fig. 5; eqn (5)) allows the determination of the corresponding enthalpy (Δ*H*°) and entropy (Δ*S*°) values (Table 1). The linearity of the association between ln(*K*_eq_) and 1/*T* indicates that enthalpy does not change appreciably within the temperature range investigated. It is noted that the Δ*H*° of the reaction of B(C_6_F_5_)_3_, *t*Bu_3_P, and CO_2_, –100.0 kJ mol^–1^, is in excellent agreement with the value of –100.4 kJ mol^–1^ obtained calorimetrically by Autrey and coworkers.^22^
3
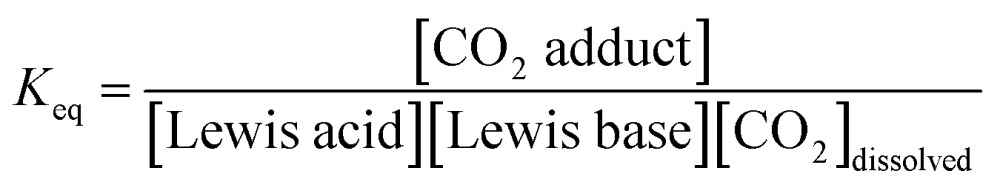

4 Δ*G*° = –*RT* ln(*K*_eq_)

5
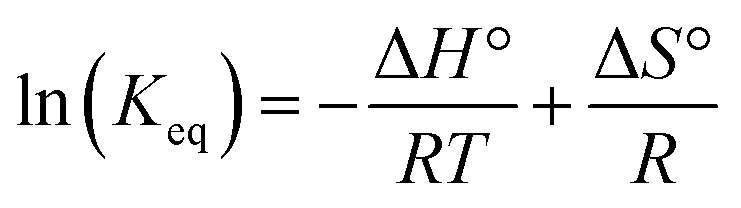



To gain further insight, theoretical calculations were performed using Density Functional Theory (DFT) at the M11/6-311G(d,p) level of theory.^23^ The optimized geometries of B(C_6_F_5_)_3_, *t*Bu_3_P, CO_2_, and *t*Bu_3_PCO_2_B(C_6_F_5_)_3_ were computed using the integral equation formalism variant of the polarizable continuum model (IEFPCM) to implicitly assess the effects of solvation by bromobenzene.^24^ Frequency calculations confirmed that each structure was at a minimum on its potential energy surface and provided partition functions from which thermodynamic parameters were computed. The reaction enthalpy obtained at this level of theory, –176 kJ mol^–1^, was significantly larger than the experimental value of –100.0 kJ mol^–1^. To further investigate this discrepancy, the calculations were carried out at the B2PLYP-D3/6-311G(d,p) level of theory.^25^ This double hybrid meta-GGA method includes an empirical long-range dispersion correction and performs well in the evaluation of main group thermochemistry.^26^ A recent study comparing the ability of M11 and B2PLYP-D3 to evaluate the chemistry of compounds for which dispersive interactions are important found the latter to consistently outperformed the former.^27^ The internal reaction energy for CO_2_ capture by the B(C_6_F_5_)_3_/*t*Bu_3_P FLP was computed to be –129 kJ mol^–1^ at this double hybrid level of theory. This value more closely approaches the experimental reaction enthalpy. In addition, if this internal reaction energy is used in combination with the reaction entropy obtained at the M11/6-311G(d,p) level of theory, then the Gibbs free energy of the reaction computed at ambient temperature is –15.4 kJ mol^–1^, in excellent agreement with the experimental value of –15.2 kJ mol^–1^.^28^

The experimentally determined thermodynamic parameters illustrate that CO_2_ binding by the two presently studied FLP systems and the previously studied ClB(C_6_F_5_)_2_/*t*Bu_3_P system^19*a*^ is less entropically favoured than that by (NI*i*Pr)_3_P, consistent with the three-component nature of the FLP reactions. On the other hand, CO_2_ binding by the FLP systems is more enthalpically favoured than that by (NI*i*Pr)_3_P, consistent with the formation of two bonds in the FLP products *versus* only one bond in the reaction with (NI*i*Pr)_3_P. Given that the phosphine (NI*i*Pr)_3_P is among the strongest of nucleophiles known to independently bind CO_2_, it is expected that this phosphine should be more efficient than simple amines, consistent with the presently described results with TMP. The present observations suggest that bidentate binding by an FLP improves the efficiency for CO_2_ capture.

Of the three systems examined, the *t*Bu_3_P/B(C_6_F_5_)_3_ FLP is most efficient at CO_2_ capture at ambient temperatures; however the phosphine (NI*i*Pr)_3_P has the most negative Δ*G*_293_ among the systems studied. Among the FLP systems, *t*Bu_3_P/B(C_6_F_5_)_3_ has a more exergonic ambient-temperature CO_2_ binding reaction than TMP/B(C_6_F_5_)_3_, but the thermodynamic parameters predict that, at elevated temperatures (347–365 K), the reaction of TMP/B(C_6_F_5_)_3_ with CO_2_ will be more exergonic. Nonetheless, these data infer that at room temperature the FLP derived from *t*Bu_3_P/B(C_6_F_5_)_3_ binds CO_2_ more effectively that the FLP derived from TMP/B(C_6_F_5_)_3_.

## Conclusions

The present study illustrates the power of the time- and labor-efficient MF platform for the qualitative and quantitative assessment of CO_2_ binding by small molecules and for the determination of the thermodynamic parameters of these reactions. These data form a quantitative basis for comparison of CO_2_ capture systems at different temperatures. The systems considered in this paper included FLPs, *t*Bu_3_P/B(C_6_F_5_)_3_ and TMP/B(C_6_F_5_)_3_, and the highly basic phosphine (NI*i*Pr)_3_P, which effectively span the range known for interactions of CO_2_ with Lewis acids and bases. The data reveal the FLP derived from *t*Bu_3_P and B(C_6_F_5_)_3_ to be the most efficient at capturing CO_2_ at ambient temperature per equivalent of CO_2_ binding unit. However (NI*i*Pr)_3_P offers a higher CO_2_ content by mass. Certainly, these data do infer that further study of FLPs in CO_2_ capture may uncover new systems that are more readily available (*i.e.* cheaper) and offer improved efficiency. To this end, we are continuing to employ this innovative MF methodology to assess and design new FLP systems for CO_2_ capture and ultimately reduction. In addition, the study of other reactions that occur at a gas–liquid interface continues. The results of these studies will be reported in due course.

## References

[cit1] Karl T. R., Trenberth K. E. (2003). Science.

[cit2] Jessop P. G., Ikariya T., Noyori R. (1995). Chem. Rev..

[cit3] Olah G. A., Goeppert A., Prakash G. K. S. (2009). J. Org. Chem..

[cit4] Bontemps S., Vendier L., Sabo-Etienne S. (2012). Angew. Chem., Int. Ed..

[cit5] Lee K. B., Beaver M. G., Caram H. S., Sircar S. (2008). Ind. Eng. Chem. Res..

[cit6] Banerjee R., Phan A., Wang B., Knobler C., Furukawa H., O'Keeffe M., Yaghi O. M. (2008). Science.

[cit7] Rochelle G. T. (2009). Science.

[cit8] Vaidya P. D., Kenig E. Y. (2007). Chem. Eng. Technol..

[cit9] Villiers C., Dognon J. P., Pollet R., Thuery P., Ephritikhine M. (2010). Angew. Chem., Int. Ed..

[cit10] Holbrey J. D., Reichert W. M., Tkatchenko I., Bouajila E., Walter O., Tommasi I., Rogers R. D. (2003). Chem. Commun..

[cit11] Mömming C. M., Otten E., Kehr G., Fröhlich R., Grimme S., Stephan D. W., Erker G. (2009). Angew. Chem., Int. Ed..

[cit12] Ashley A. E., Thompson A. L., O'Hare D. (2009). Angew. Chem., Int. Ed..

[cit13] Declercq R., Bouhadir G., Bourissou D., Légaré M.-A., Courtemanche M.-A., Nahi K. S., Bouchard N., Fontaine F.-G., Maron L. (2015). ACS Catal..

[cit14] Courtemanche M.-A., Larouche J., Légaré M.-A., Bi W., Maron L., Fontaine F.-G. (2013). Organometallics.

[cit15] Weicker S. A., Stephan D. W. (2015). Chem.–Eur. J..

[cit16] Wünsche M. A., Mehlmann P., Witteler T., Buß F., Rathmann P., Dielmann F. (2015). Angew. Chem., Int. Ed..

[cit17] (b) Mehlmann P., Mück-Lichtenfeld C., Tan T. T. Y. and Dielmann F., Chem.–Eur. J., 10.1002/chem.201604971. 27779340

[cit18] Bishnoi S., Rochelle G. T. (2000). Chem. Eng. Sci..

[cit19] Voicu D., Abolhasani M., Choueiri R., Lestari G., Seiler C., Menard G., Greener J., Guenther A., Stephan D. W., Kumacheva E. (2014). J. Am. Chem. Soc..

[cit20] Voicu D., Stephan D. W., Kumacheva E. (2015). ChemSusChem.

[cit21] Welch G. C., San Juan R. R., Masuda J. D., Stephan D. W. (2006). Science.

[cit22] Karkamkar A., Parab K., Camaioni D. M., Neiner D., Cho H., Nielsen T. K., Autrey T. (2013). Dalton Trans..

[cit23] Krishnan R., Binkley J. S., Seeger R., Pople J. A. (1980). J. Chem. Phys..

[cit24] Tomasi J., Mennucci B., Cammi R. (2005). Chem. Rev..

[cit25] Grimme S. (2006). J. Chem. Phys..

[cit26] Goerigk L., Grimme S. (2011). J. Chem. Theory Comput..

[cit27] Goerigk L. (2015). J. Phys. Chem. Lett..

[cit28] Computed thermochemical parameters for the binding of CO_2_ by (NI*i*Pr)_3_P afforded values significantly greater than those reported here (ref. 17*b*). Preliminary follow-up computations suggest that a monotonic relationship between the amount of Hartree–Fock exchange included and the computed internal energy of reaction for CO_2_ binding to (NI*i*Pr)_3_P. While we do not as of yet have an explanation for this observation, it is noted that the present computations show better agree with the experimental values derived herein

