# Supplementary material for: Quantifying the efficiency of CO2 capture by Lewis pairs
Source: Chem Sci. 2017 Feb 20;8(4):3270–5. doi: 10.1039/c6sc05607e (PMC5424443; doi:10.1039/c6sc05607e)
Supplement: Supplementary file 1 [file SC-008-C6SC05607E-s001.pdf]

Electronic Supplementary Material (ESI) for Chemical Science.
This journal is © The Royal Society of Chemistry 2017

Supporting Information

Quantifying CO_2 Capture by Lewis Pairs

Jay J. Chi, Timothy C. Johnstone, Dan Voicu, Paul Mehlmann, Fabian Dielmann, Eugenia Kumacheva*, and Douglas W. Stephan*

Table of Contents

General Methods 2
Microfluidic Experimental Setup 2
Data Acquisition and Analysis 4
Pressure Drop Calculation Along Microfluidic Channels 4
Computational Details 5
Variable Temperature Microfluidic Experiments 6
Thermodynamic Parameters 7
Reaction of $(NIiPr)_3P$ and $B(C_6F_5)_3$: 8
References 9

General Methods

Both tri-tert-butylphosphine (tBu_3P) and 2,2,6,6-tetramethylpiperidine (TMP) were purchased from Sigma-Aldrich and purified by distillation. Tris(pentafluorophenyl)borane, $B(C_6F_5)_3$, was obtained from Boulder Scientific and purified by sublimation under reduced pressure at 90 °C prior to use. Tris(1,3-diisopropyl-4,5-dimethylimidazolin-2-ylideamino) phosphine, (NI*i*Pr)$_3$P, was synthesized as described elsewhere.[1] Bromobenzene was purchased from Caledon (Canada), distilled, and stored over 3 Å molecular sieves. All reagent solutions were dissolved in bromobenzene to various concentrations ranging from 0 to 200 mM. These solutions were prepared in a nitrogen-filled glovebox (0.6-1.5 ppm O_2) to avoid oxygen and moisture exposure, which would poison the Lewis acid-base or FLP systems.

Microfluidic Experimental Setup

All solutions containing Lewis acid-base reagents dissolved in bromobenzene were supplied to a microfluidic reactor, R150.332.2 from Micronit Microfluidics (Netherlands), depicted in Figure S1 below. The glass microreactor is comprised of two inlets that converge to form a Y-junction and one outlet. Prior to experiments, the reactor was dried overnight in an oven at 100 °C to remove traces of moisture. The microreactor, clamped and secured within a custom-made acrylic manifold containing embedded Nanoport connections (IDEX Health Science, USA), was purged with dry CO_2 gas (Grade 4.0, Linde) for 10 minutes prior to the start of experiments at 2 psig to remove trace air content. Specific concentrations of desired reagents were supplied to the MF reactor via two glass syringes coupled to a syringe pump (PHD2000, Harvard Apparatus). In order to account for reagent dilution upon mixing, concentrations would be double the on-chip concentration; for instance, to study a 50 mM FLP solution, each syringe

would be loaded with 100 mM of Lewis acid and 100 mM of Lewis base solutions and loaded to the reactor at 2.5 μL/min. Consequently, the total on-chip volumetric flow rate of reagent solution was 5 μL/min. Other details with regards to the general experimental setup are described elsewhere.[2] Data were collected using 1.25x magnification on an inverted microscope (Olympus IX71, Olympus Canada) to image CO_2 and FLP segmented flow through the microreactor in our desired field of view shown again in Figure S1 below. A Matlab-based program was used to automatically measure CO_2 plug length and plug position in a specific region of interest as described elsewhere.[3] For all reduced and elevated temperature experiments, methodology similar to that described elsewhere[3] was followed.

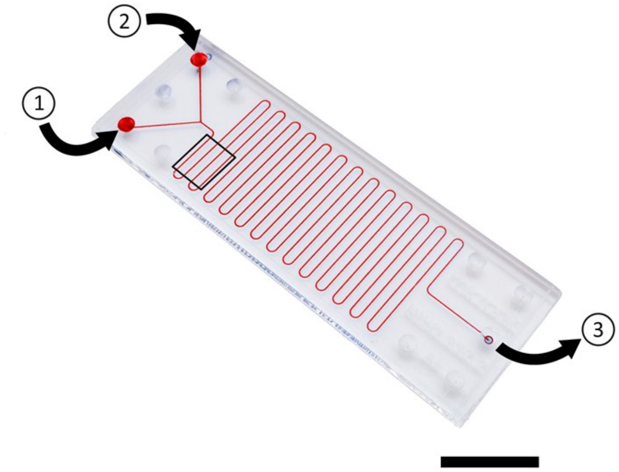

Figure S1. An image of the microfluidic reactor, which is comprised of two inlets, (1) and (2), and one outlet (3). Red dye was used to accentuate the channel. The microchannel had the width, height, and length of 150 μm, 150 μm, and 30.4 cm, respectively with a total volume of 6 μL. A representative region of interest used for data analysis is outlined with a black square. The change in length of CO_2 bubbles outside this examined field of view was not analyzed, which led to gaps in experimental data points between 1.5 to 2 s (Figure 2). The scale bar is 500 μm.

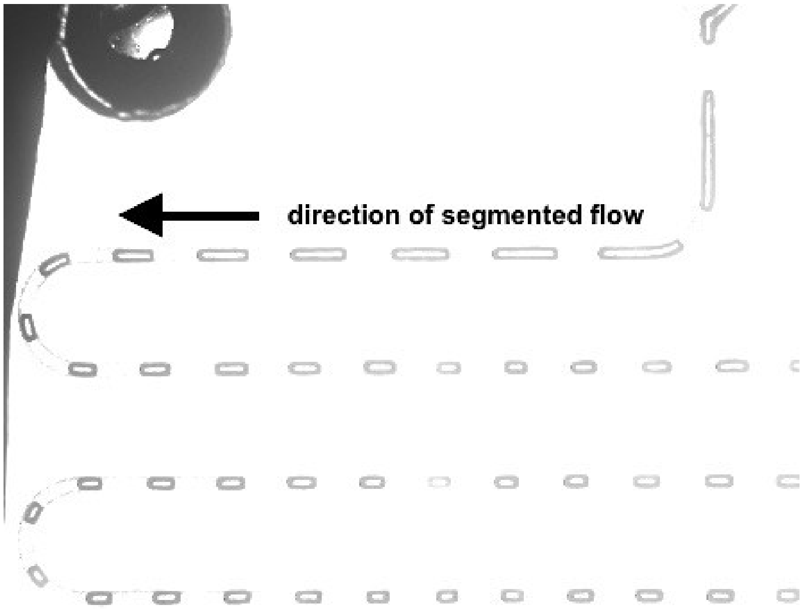

Figure S2. Optical micrograph of the region of interest, which depicts the shrinking gaseous plugs (dark segments) and the liquid slugs (gaps between segments) taken at 1.25x magnification on an inverted microscope.

Data Acquisition and Analysis

For each concentration of reagent, three sequences of 300 images were acquired in the field of view (Figure S1) with at least 4000 gas bubbles to 8000 bubbles detected. Using a Matlab-based program code described elsewhere,[2] the gas plug length, $L_{P,}$ and slug length, L_S, and velocity of CO_2 plugs through the MF rector at different locations were calculated. The average value of L_P at a particular location and velocity were determined and standard deviation was calculated. This Matlab program does not detect plugs that flow through microchannel bends, which leads to gaps in experimental points (e.g. at 1.5 s in Figure 2). Specific aspects and equations employed in experimental data analysis are described in detail elsewhere.[2]

Pressure Drop Calculation Along Microfluidic Channels

Although CO_2 is supplied at a constant pressure of 118.5 kPa to the microreaction, the actual pressure found at the Y-junction, P_0, will be slightly lower due to a pressure drop along the microchannel. The initial pressure at the Y-junction was determined to be:

$$P_o = P_{out} + \left(\frac{dP}{dX}\right)L \quad (1)$$

where P_{out} represents the atmospheric pressure at the outlet of the MF reactor, (dP/dX) represents the pressure drop along the microchannels, and finally L is the total channel length stemming from the Y-junction to the outlet of the reactor. The pressure drop along the channel was calculated as:[2,3]

$$\frac{\Delta P}{X} = \beta_L f_{slug}\left(\frac{2\rho {U_P}^2}{D_h}\right) \tag{2}$$

$$f_{slug} = \frac{16}{Re}\left[1 + 0.17\frac{w}{L_S}\left(\frac{Re}{Ca}\right)^{0.33}\right] \tag{3}$$

where ΔP/X represents the pressure drop along the channel, β_L represents the liquid volume fraction in the channel from the Y-junction to the outlet, f_{slug} represents the friction factor for segmented flow, Ca is the capillary number, Re is the Reynold's number, and p is the liquid viscosity.

Computational Details

All calculations were carried out using Gaussian 09, rev. D.[4] This work was made possible by the facilities of the Shared Hierarchical Academic Research Computing Network (SHARCNET:www.sharcnet.ca) and Compute/Calcul Canada. Geometry optimizations were performed using, as input geometries, either coordinates deposited in the Cambridge Structural Database or coordinates generates by hand with GaussView 5.0.[5] Structures were initially optimized in the gas phase at the M11/6-31g(d,p) level of theory, and then subsequently at the M11/6-311g(d,p) level with bromobenzene implicit solvation.[6, 7] Finally, the geometry was optimized at the M11/6-311g(d,p) level with implicit bromebenzene solvation, strict convergence criteria (opt=VeryTight) and a large grid (UltraFineGrid). Implicit solvation was implemented using a solvent cavity reaction field. Frequency calculations were used to confirm that geometries optimized to local minima on their respective potential energy surfaces and to obtain thermochemical analyses. Reaction enthalpies were computed by taking the difference of the sums of the thermally-corrected enthalpies of the products and reactants. Reaction entropies and Gibb's free energies were computed analogously. The CO_2 sequestration by the BCF/tBu_3P FLP was also explored at the B2PLYP/6-311g(d,p) level of theory. Single point energy calculations were performed and the resulting MP2-corrected energies were taken as an estimate of ΔH_{rxn}.[8] The reaction stoichiometries used in the thermochemical analyses are those depicted in Scheme 1. The enthalpies, energies, and Gibbs free energies of reaction are collected in Table S1.

Table S1. Computed thermodynamic parameters.

**Level of Theory:** M11/6-311g(d,p)			
**Reaction system**	**ΔH_{298} (kJ/mol)**	**ΔS_{298} (J/mol K)**	**ΔG_{298} (kJ/mol)**
BCF/*t*Bu_3P	-175.959	-380.568	-62.4922
BCF/TMP	-136.98	-369.832	-26.7119
P(NI*i*Pr)$_3$	-82.9554	-133.156	-43.2551

**Level of Theory:** B2PLYP/6-311g(d,p)		
**Reaction system**	**ΔE (kJ/mol)**	**ΔG_{298} (kJ/mol)[a]**
BCF/*t*Bu_3P	-128.769	-15.4108

[a] Calculated under the approximation that $\Delta H = \Delta E$ and using the ΔS from the M11 calculations.

Variable Temperature Microfluidic Experiments

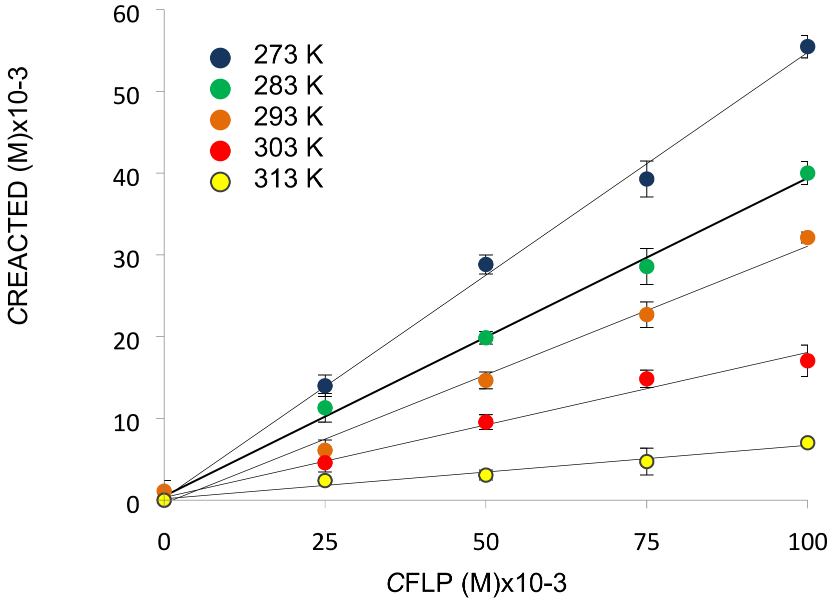

Figure S3. Variation in equilibrium reaction-induced chemical CO_2 uptake, plotted as a function of the initial concentration of the FLP consisting of BCF and TMP at various temperatures: 273 K, 283 K, 293 K, 303 K, and 313 K.

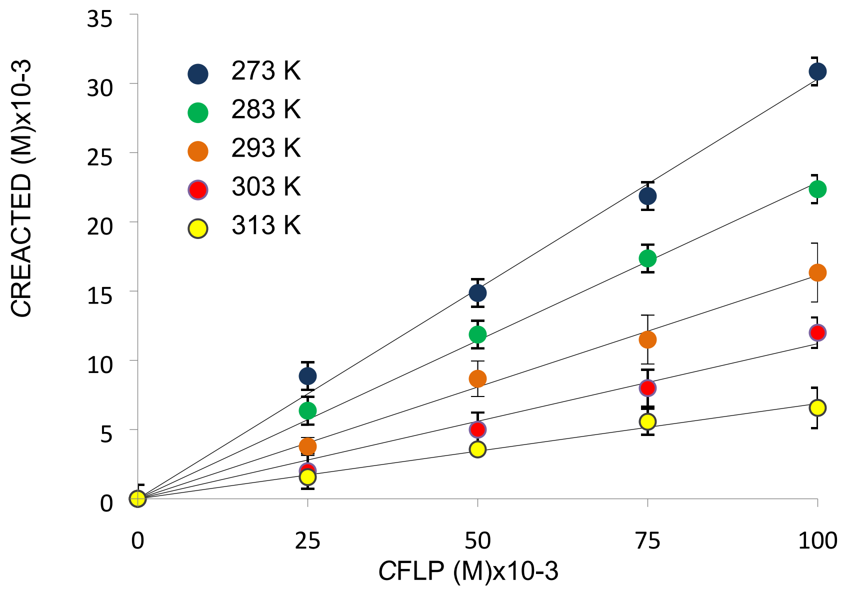

Figure S4. Variation in equilibrium reaction-induced chemical CO_2 uptake, plotted as a function of the initial concentration of the FLP consisting of P(NI*i*Pr)$_3$ at various temperatures: 273 K, 283 K, 293 K, 303 K, and 313 K.

Thermodynamic Parameters

Table S2. Thermodynamic Parameters for the BCF/*t*Bu$_3$P Reaction with CO_2.

T(K)	273	283	293	303	313
ΔH (kJ mol^{-1})	-100.0	-100.0	-100.0	-100.0	-100.0
ΔS (J mol^{-1} K^{-1})	-288.9	-289.4	-289.3	-287.5	-289.8
ΔG (kJ mol^{-1})	-21.1	-18.1	-15.2	-12.9	-9.3

Table S3. Thermodynamic Parameters for the BCF/TMP Reaction with CO_2.

T(K)	273	283	293	303	313
ΔH (kJ mol^{-1})	-73.8	-73.8	-73.8	-73.8	-73.8
ΔS (J mol^{-1} K^{-1})	-204.2	-205.9	-205.4	-204.1	-205.1
ΔG (kJ mol^{-1})	-18.0	-15.5	-13.6	-11.9	-9.6

Table S4. Thermodynamic Parameters for the P(NI*i*Pr)$_3$ Reaction with CO_2.

T(K)	273	283	293	303	313
ΔH (kJ mol^{-1})	-29.1	-29.1	-29.1	-29.1	-29.1
ΔS (J mol^{-1} K^{-1})	-31.5	-31.3	-30.8	-31.0	-31.6
ΔG (kJ mol^{-1})	-20.5	-20.2	-20.0	-19.7	-19.2

Reaction of $(NIiPr)_3P$ and $B(C_6F_5)_3$:

$B(C_6F_5)_3$ (25.0 mg, 0.05 mmol) was added to a solution of $P(NI\textit{i}Pr)_3$ (30.0 mg, 0.05 mmol) in fluorobenzene at room temperature. The NMR analysis of the reaction mixture (Figure 1-4) indicate the formation of several species. A major product is thought to result from the attack of $(NIiPr)_3P$ at the perfluorobenzene ring of $B(C_6F_5)_3$.

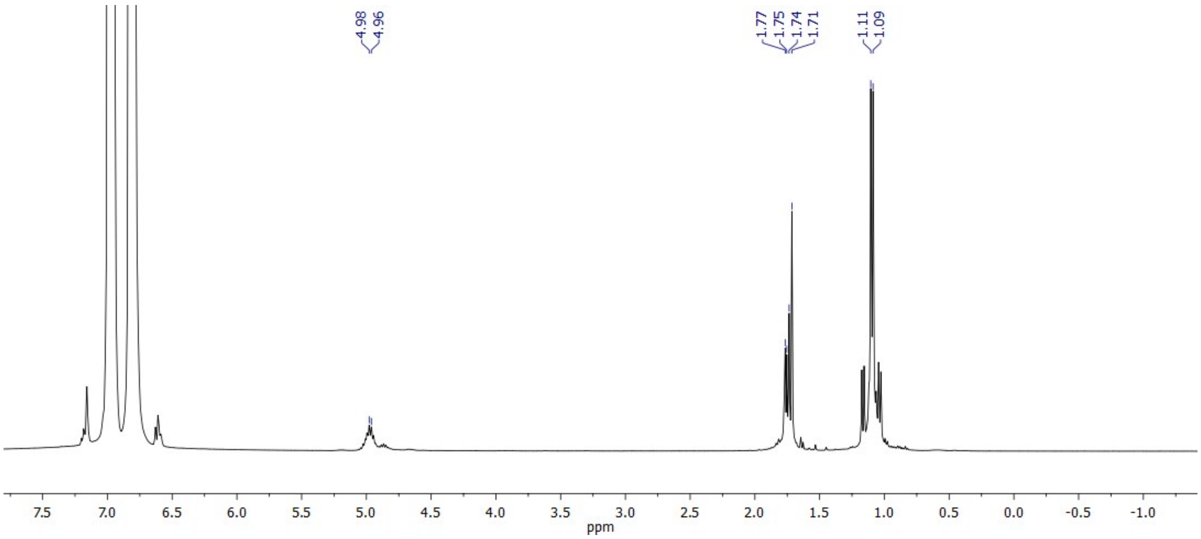

Figure S5: ^{1}H NMR

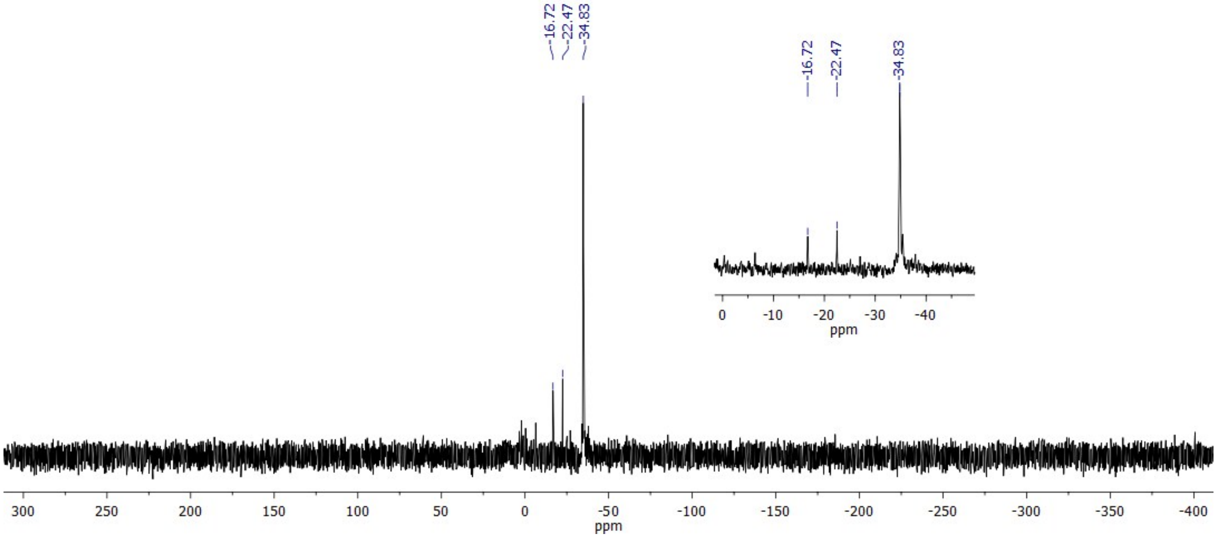

Figure S6: ^{31}P NMR

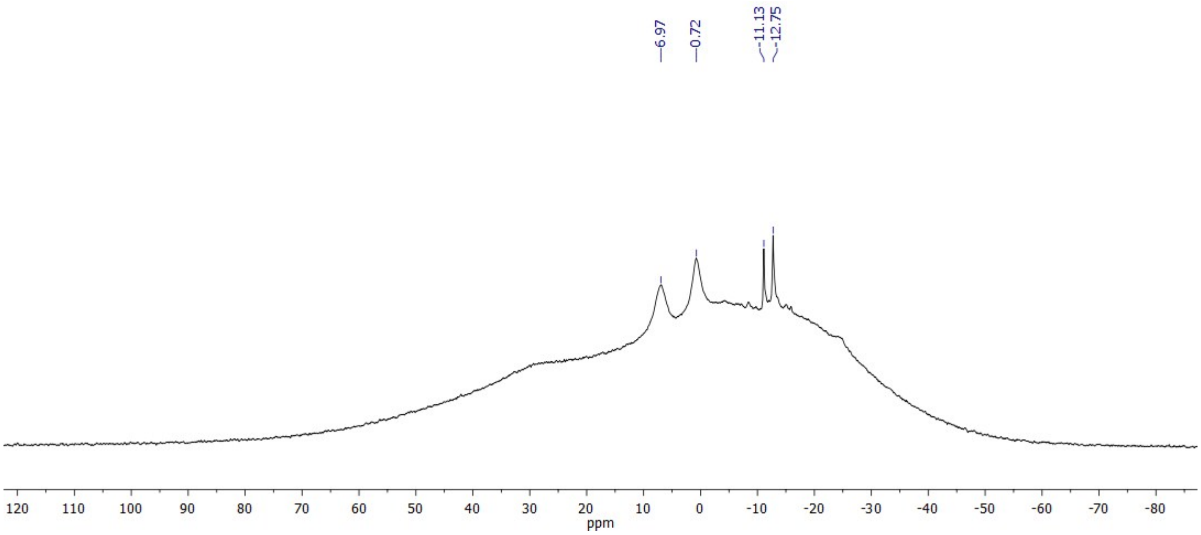

Figure S7: ^{9}B NMR

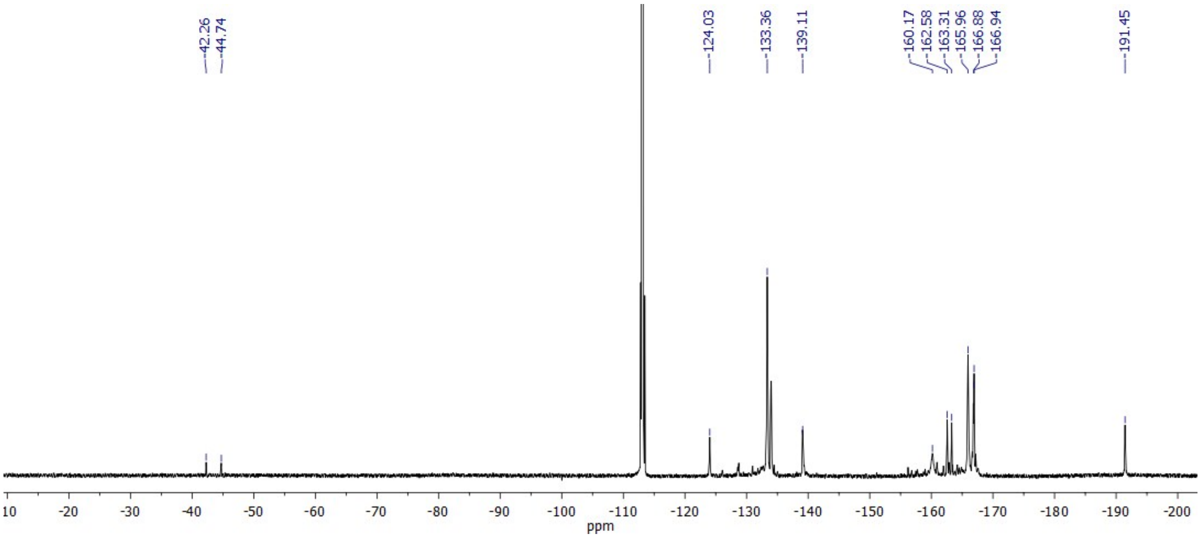

Figure S8: ^{19}F NMR

References

1. F. Buß, P. Mehlmann, C. Mück-Lichtenfeld, K. Bergander and F. Dielmann, *J. Am. Chem. Soc.*, 2016, **138**, 1840-1843.
2. D. Voicu, M. Abolhasani, R. Choueiri, G. Lestari, C. Seiler, G. Menard, J. Greener, A. Guenther, D. W. Stephan and E. Kumacheva, *J. Am. Chem. Soc.*, 2014, **136**, 3875-3880.

3. D. Voicu, D. W. Stephan and E. Kumacheva, *ChemSusChem*, 2015, **8**, 4202-4208.
4. G. W. T. M. J. Frisch, H. B. Schlegel, G. E. Scuseria, M. A. Robb, J. R. Cheeseman, G. Scalmani, V. Barone, B. Mennucci, G. A. Petersson, H. Nakatsuji, M. Caricato, X. Li, H. P. Hratchian, A. F. Izmaylov, J. Bloino, G. Zheng, J. L. Sonnenberg, M. Hada, M. Ehara, K. Toyota, R. Fukuda, J. Hasegawa, M. Ishida, T. Nakajima, Y. Honda, O. Kitao, H. Nakai, T. Vreven, J. A. Montgomery Jr., J. E. Peralta, F. Ogliaro, M. Bearpark, J. J. Heyd, E. Brothers, K. N. Kudin, V. N. Staroverov, T. Keith, R. Kobayashi, J. Normand, K. Raghavachari, A. Rendell, J. C. Burant, S. S. Iyengar, J. Tomasi, M. Cossi, N. Rega, J. M. Millam, M. Klene, J. E. Knox, J. B. Cross, V. Bakken, C. Adamo, J. Jaramillo, R. Gomperts, R. E. Stratmann, O. Yazyev, A. J. Austin, R. Cammi, C. Pomelli, J. W. Ochterski, R. L. Martin, K. Morokuma, V. G. Zakrzewski, G. A. Voth, P. Salvador, J. J. Dannenberg, S. Dapprich, A. D. Daniels, O. Farkas, J. B. Foresman, J. V. Ortiz, J. Cioslowski and D. J. Fox, Gaussian 09, Revision D.01, (2013) Gaussian, Inc., Wallingford, CT.
5. R. K. Dennington, T.; Millam, J., *GaussView, Version 5*, Semichem Inc., Shawnee Mission, KS.
6. W. J. Hehre, R. Ditchfield and J. A. Pople, *J. Chem. Phys.*, 1972, **56**, 2257-2261.
7. R. Peverati and D. G. Truhlar, *J. Phys. Chem. Lett.*, 2011, **2**, 2810-2817.
8. C. M. Mömming, E. Otten, G. Kehr, R. Fröhlich, S. Grimme, D. W. Stephan and G. Erker, *Angew. Chem. Int. Ed.*, 2009, **48**, 6643-6646.
